# The transition of assessing health technologies to social interventions in Sweden

**DOI:** 10.1017/S0266462324000606

**Published:** 2024-11-29

**Authors:** Sophie Söderholm Werkö, Titti Mattsson, Sofia Tranæus, Pernilla Östlund, Knut Sundell

**Affiliations:** 1 The Swedish Agency for Health Technology Assessment and Assessment of Social Services (SBU), Stockholm, Sweden; 2 Faculty of Law Health Law Research Centre, Lund University, Lund, Sweden; 3 Health Technology Assessment-Odontology (HTA-O), Faculty of Odontology, Malmö University, Malmö, Sweden; 4 Department of Social Work and Criminology, University of Gävle, Gävle, Sweden

**Keywords:** evidence-based policy, systematic literature review, health technology assessment, social intervention assessment

## Abstract

Since the 1970s the Swedish government has been promoting social work based on research into methods which work in practice for practitioners and patients. In 2015, the Swedish Agency for Health Technology Assessment (SBU), a government agency instigated in 1987, was commissioned to expand its remit, to review empirical research on social work interventions and to disseminate the results to stakeholders. SBU was then renamed The Swedish Agency for Health Technology Assessment and Assessment of Social Services (SBU). This article describes the fusion of health technology assessment (HTA) and Social Intervention Assessment (SIA), including advantages and challenges.

## Introduction

This article gives an account of initiatives by the Swedish government intended to promote evidence-based social work. In 2015, the mandate of the Swedish Agency for Health Technology Assessment (HTA) was extended to include social services: Now renamed The Swedish Agency for Health Technology Assessment and Assessment of Social Services (SBU). This article describes the integration of social intervention assessment (SIA) into a national HTA organization. The description of this transformation may be of particular interest now given the current growing interest in evidence-based social service interventions. The context and organization of SBU are presented first, followed by a description of the integration of HTA and SIA.

## The Swedish context

If one country were to be singled out as the archetypal welfare state, Sweden would be among the top contenders. The Swedish model has a universal approach, meaning that at some time during their lifespan, the benefits created by tax transfers are shared by everyone, without individual means testing (1). Sweden has extensive social services. Of the population of 10 million, approximately 800,000 receive social service support annually, for example, those with functional disabilities, alcohol and drug misuse problems, children, and youth in need of protection and care, the elderly and the poor (2). The level of funding for social services is not as high as for health care, but it is still significant: SEK 275 billion (approx. €25.9 billion or US$27.3 billion) and 368 billion (approx. €34.6 billion or US$36.5 billion), respectively in 2022. The delivery of health services is primarily the responsibility of the 21 regions (former county councils), while social services, including aged care, are managed locally by Sweden’s 290 municipalities.

Although the social services system deals with a large proportion of the Swedish population, at a national level the services have not generally been based on systematic research intended to distinguish between effective procedures and those which are ineffective or even harmful. There are hundreds of social work interventions (methods, treatments, and services) in use, but few have been scientifically evaluated. An inventory of the child welfare services currently in use in Sweden identified 102 different interventions, of which only nine were well-supported by research, according to the California Evidence-Based Clearinghouse, that is at least two rigorous randomized controlled trials found the interventions to be superior to an appropriate comparison (3).

## Evidence-based policy in social services

The Swedish government has a long history of promoting empirical social research (4; 5). In 1977, social work was introduced as an academic discipline in Sweden, with full academic rights to PhD training and other scholarly activities. A special research council for social research and development was also established at the time. However, the focus of the academy was never on evaluation of interventions.

In 1993, the Center for Evaluation of Social Services was established, and in 2004 transformed into the Institute for Evidence-Based Social Work Practice. One of the tasks of the institute was to synthesize social work interventions in Systematic Reviews. This marked a new strategy by national policymakers to bridge the gap between evidence and practice in social services. In 2010, the Institute was brought under the auspices of the National Board of Health and Welfare (NBHW). At the same time, the NBHW was reorganized to better accommodate evidence-based policy and evidence-based practice (5). The work of systematic reviews continued within a unit at the NBHW.

In 2015, the government commissioned SBU to conduct systematic reviews of empirical research into social work interventions. One motive (6) was to utilize SBU’s long experience of systematic reviews, in order to enhance the quality of such reviews within social services. Another motive was to strengthen the links between health care and social services and to encourage interdisciplinary activity. A third was to delineate the task of producing evidence from the task of formulating guidelines. A fourth motive was to foster commitment of social services to evaluation research, by involving social service experts in SBU’s internal review work. In accordance with the expanded mandate, SBU was renamed The Swedish Agency for Health Technology Assessment and Assessment of Social Services.

## The Organization

SBU is an independent government agency within the Ministry of Health and Social Affairs. It is fully funded by the government and its role is to evaluate scientific evidence supporting both new and established interventions and technologies within health (since 1987), dental care (since 1999), the working environment (since 2011) and social services, including services for people with certain functional disabilities (since 2015). SBU is also Sweden’s international contact on issues related to the evaluation of interventions (7).

SBU is a HTA organization. Apart from systematic reviews on the benefits and harmful effects of methods (interventions, services, practices, and technologies), assessments also include economic and ethical analyses. Where appropriate, organizational and legal aspects are also included in the reviews. As well as a synthesis of the effects of interventions, the review can cover diagnosis and risk assessment, as well as prevention and risk factors. Each assessment addresses a specific question or topic and can serve as support for decision-makers in medical or social services. While both quantitative and qualitative studies can be included, to date most have had a quantitative focus.

Today most projects are initiated by the Ministry of Health and Social Affairs or by government agencies, especially the NBHW, which is the major agency producing guidelines for health and social services.

SBU is governed by a Director General. SBU is supported by a Scientific Advisory Committee, comprising 20 senior academic experts within the primary SBU research fields. Other important stakeholder groups which to a varying extent participate in the work of SBU include clinical experts, patient and client advocates, research councils, and representatives of academia. All external experts or stakeholders have to fill out a conflict of interest declaration which must be approved before they can participate in any SBU projects. Systematic interaction with patients, clients, and users is important, as it is believed that this will increase both the relevance of the products and their use. Regular interaction with the scientific community outside SBU is also important, not only for input but also to guarantee that the work of SBU will be of the highest quality. SBU currently has a staff of around 100, half of whom have a PhD degree (2022). In 2021, the budget was SEK 100,000,000 (approx. €9,440,000 or US$10,173,000).

The review work is undertaken by project teams, comprising a number of external researchers with expert knowledge of the issue to be investigated and SBU staff who guarantee that systematic reviews comply with Cochrane and Campbell collaboration standards. The staff comprises a project manager, a scientific information specialist (librarian), a health economist, and administrative personnel. The work is guided by a handbook (8) and coding templates. There are numerous intermediary steps intended to ensure high quality. For instance, SBU assessments undergo several review steps by an internal quality assurance committee, the Scientific Advisory Committee, as well as contracted external expert reviewers. The report is then published on the SBU public Website. SBU is not a policymaker, but produces evidence to support policy and decision-making in the fields of health care and social services.

## Publications

Over the years, SBU has diversified its number of products, from initially one type of product, the systematic review, to currently six types (Table 1). The products also differ in the length of time of production, for example, due to the scope of the research question and availability of scientific publications. The first product is *the SBU assessment report*: a comprehensive, systematic assessment of the available scientific evidence. The certainty of the evidence for each finding is systematically reviewed and graded. Full assessments include economic, social, and ethical impact analyses, as well as client/patient participation. These reports are the most comprehensive of SBU’s products. The target groups are national, regional, and local decision-makers.

**Table 1.** SBU’s products

	Assessment	Policy support	Evidence map	Commentary	Evidence gaps	Enquiry response
Systematic review	X	X	X	X	X	X
Relevance	X	X	X	X	X	X
Risk of bias	X	X	X	X	X	X
Grading (GRADE)	X	(X)			X	
External expertise	X	X	X	X	X	(X)
Client/patient participation	X			(X)	(X)	(X)
Economics	X	(X)		(X)		(X)
Ethics	X	(X)		(X)		(X)
Inventory of Swedish practice	(X)					
Duration (months)	18–24	6–9	12	3–6	-	2–3
Target group	Decision-makers	Government Agencies	Scientific community	Decision-makers	Scientific community	Decision-makers

*The SBU policy support report* is another form of publication which identifies and presents the scientific evidence available to support policy and decision-making by other government agencies, including the development of national guidelines. In contrast to the SBU Assessment, SBU leaves it to the commissioner to include economics, ethics, and Swedish praxis.

*The SBU commentary report* summarizes and examines selected high-quality systematic reviews published elsewhere (e.g., by other agencies or by academics). External experts within the relevant topic of each assessment help the staff to set the results in a Swedish context. When high-quality reviews are available, this is an efficient way of distributing knowledge relevant to Swedish stakeholders.

*The SBU evidence map* evaluates the quality of systematic reviews in a specific field (e.g., financial aid) to reliably identify evidence and gaps in scientific knowledge. It gives a broad perspective over a large area and could be a starting point for further assessments. The target groups for this product are both professionals within various fields and the scientific community.

The fifth product, *Evidence Gaps*, also targets the scientific community. This is a by-product of the SBU assessments, policy supports, and evidence maps, produced with the aim of reducing research waste (9). When methods are identified for which there is no conclusive systematic review of benefits and harmful effects, or if a systematic review of high standard concludes that there is a need for primary research, the evidence gap is entered into a database. The intention is that the database will be used by research funders in calls for new research and this has also occurred. Taking the Evidence Gaps one step further is SBU’s Prioritizations of scientific evidence gaps, using a method developed by the James Lind Alliance in Great Britain, whereby a broad range of stakeholders is actively engaged in the prioritization process, including healthcare and social service providers as well as service users and their families (10).

The sixth and final publication type is *the Enquiry Service* which is intended to support the regional needs of social services and healthcare in Sweden. Swedish healthcare professionals or social service providers can ask SBU to assess specific questions about the efficacy of methods. Responses from the Enquiry Service are reports answering such specific questions and are based on systematic literature searches in a limited number of databases. The quality of the included systematic reviews is assessed, but not the individual studies comprising the review.

## Dissemination

SBU is commissioned to publish research results in Swedish. SBU Assessments are generally not sent to Swedish Universities. However, the engaged experts within the projects, usually affiliated with or employed by a University, contribute with this task. To enhance international sharing of the results, an English summary of each SBU Assessment is published on the Website. The project group is also encouraged to undertake dual publication, in English and Swedish. Approximately a quarter of these publications are published in international peer-reviewed journals. Moreover, the English summaries of all SBU Assessments and SBU Evidence Maps are published in PubMed. Table 2 presents the production of the different types of reports within the fields of healthcare and social services, respectively. Approximately two thirds of the publications concern the health sector. Records of the number of visitors to the SBU Website do not differentiate between those visiting the health or social services sectors. However, the number of visitors has been increasing constantly and has more than doubled since 2016.

**Table 2.** SBU reports published 2016–2022

	2016		2017		2018		2019		2020		2021		2022		All	
	HC	SS	HC	SS	HC	SS	HC	SS	HC	SS	HC	SS	HC	SS	HC	SS
SBU Assessment	10	1	3	1	10	3	2	1	2	1	9	2	6	4	42	13
SBU Policy support	4	0	14	1	8	1	5	1	8	1	5	0	4	1	48	5
SBU Evidence map	2	0	3	2	1	1	0	3	1	0	3	0	0	0	10	6
SBU Commentary	14	4	3	0	10	4	5	7	5	5	7	4	5	5	49	29
Evidence gaps	277	47	333	46	191	145	213	31	284	33	124	31	227	25	1649	358
SBU Enquiry service	30	3	26	5	23	12	42	19	21	19	34	9	18	16	194	83
Visitors to Web site	615,000		615,000		726,000		872,000		1,090,000		1,617,000		1,586,000			

HC= Healthcare; SS= Social services.

After 28 years of supporting the healthcare sector with reports, SBU is well-known and well-respected among clinicians and stakeholders in Sweden. In contrast, SBU is new to the field of social services, with fewer publications and no established reputation in the field. Initially, this makes the dissemination and impact of the reports a challenge. Compared to the healthcare system, there are few specialists social work associations in Sweden which should function as intermediaries in disseminating SBU reports. Another challenge is that historically the basic training of social workers has not been well adapted to evidence-based practice. Social work training in Sweden is gradually transitioning towards a more evidence-based practice approach. Because of this, it was decided initially to develop a relevant national social work network.

The short-term aim of this network is to strive for a common methodology to grade the quality of evidence and strength of recommendations. The long-term aim is to support the establishment of regional units, which will produce their own systematic reviews, relevant to local needs. The representatives are mainly regional research and development managers, a group well-suited to promote evidence-based practice in their regions.

Furthermore, workshops have been set up by SBU, targeting (1) the end-users of research; (2) social services researchers; and (3) government agency staff. This effort has attracted considerable interest, indicating that these initiatives meet a fundamental need. Of the end-users, one group has been the focus because of their potential importance: organizational development specialists. These social workers support both managers, by providing aggregated data (e.g., compiling client flow information) and case workers, by providing advice on choices of interventions. Until the pandemic in 2020, 150 organizational development specialists, out of approximately 900, had participated in a 5-day workshop on evidence-based social work, focusing on searching for, assessing the quality of, and communicating intervention research.

There are few systematic reviews by Swedish academics in social work. In order to increase the number of reports, all university social work departments and units of research and development have been offered a 2-day workshop on conducting systematic reviews. SBU has arranged twelve such workshops, covering most university faculties and training more than 180 researchers. A questionnaire shows that 22 percent of the participants are interested in close collaboration with SBU and 10 percent had conducted a systematic review after the course.

The last targeted group, government agency staff, has undergone 1-day training in the assessment methodology applied by SBU. The main objective has been to support the use of a practical, transparent approach to grading the quality of evidence and the strength of recommendations. Within a year of the start, 150 staff members of ten government agencies and representatives of the Ministry of Health and Social Affairs had taken the course.

With time it became apparent that the increased interest in the methodology of systematic reviews was very time-consuming for SBU staff. Together with the need for alternatives to reach out to society during the pandemic, SBU decided to produce short films describing various parts of the methodology of systematic reviewing. The first six films attracted the interest of 6,500 viewers during the first year of 2022. Another short film, about the meaning of evidence, has attracted 12,300 viewers since publication in 2019.

One of the latest novelties introduced by SBU is a list of *What works*, that is interventions identified in systematic reviews as effective. The information in this type of product is structured according to PICO; that is population, intervention, comparative condition, and outcome measures, together with the effects, are described in plain language. To date, the web page includes 38 interventions from different areas in social services. One year after the launch, it has had 4,800 visitors.

Despite such concerted efforts and activities, dissemination of SBU’s products and the attitude towards evidence-based practice is an ongoing challenge.

## Adoption and adaptation

When SBU was commissioned to include assessment of social services, there were well-tested procedures and routines from the health sector which could also be applied to social services.

Initially, there was some concern by SBU that there would be a lack of high-quality social services research. This was also the case in two of the first 9 reviews (11; 12). In itself, this was not surprising because similar “empty” reviews also occur with respect to healthcare interventions. Hopefully, the increased publication rate of primary research will reduce the risk of “empty” reviews. This is illustrated by the example of parent management training to reduce disruptive child behavior. Ten years ago, a systematic review produced inconclusive results (13), whereas recent systematic reviews report this to be effective (14).

Another concern was that social welfare interventions are more complex and heterogeneous than healthcare interventions because social welfare populations often have multiple problems and there is a lack of consensus as to the generally accepted best treatment. Although it is debatable whether healthcare interventions are equally hindered by a population with multiple problems and complex interventions, there are few generally accepted best treatments in social welfare and thus a risk of merging different control groups. Although this may constitute a problem, to date it has not prevented SBU and other organizations such as Campbell collaboration or the Evidence for Policy and Practice Information and Co-ordinating Centre (EPPI-center) from synthesizing research on social services interventions.

A third concern was that results would not easily be extrapolated from international research to the Swedish context because of the heterogeneity of clients, providers, and settings. Although the issue of transportability seldom arises in systematic reviews, some reports indicate that the results of interventions can be extrapolated (e.g., 14) while others claim the opposite (15). To date, SBU’s reviews have not raised concerns about transportability. Hopefully, the rapid increase in Swedish randomized controlled trials (16) will improve the potential to test whether or not transportability is a problem.

There was also some initial concern that it would be difficult to recruit scientific experts to projects because Swedish social services research has not focused on intervention research. However, while recruitment of experts has not been an issue, most of the experts are sourced from related disciplines, such as psychology, public health, and the caring sciences.

There are two areas in which the procedures and routines from healthcare give insufficient guidance for social welfare reviews. The first is economic evaluations of social service interventions, an important area because of its potential to improve resource allocation. Economic analyses are complicated because several different outcome measures are reported in studies and there is no commonly accepted generic measure of well-being (corresponding to QALY in health economic evaluations). Furthermore, costs often occur in different sectors, making registration time-consuming: The costs are borne by various providers and are incurred at different time points.

The second area in which there is insufficient guidance from the healthcare sector is the search for all relevant literature on social services. This is because research on social welfare is multidisciplinary, and as a consequence, terms are sometimes ambiguous, differently defined and changing. Another problem is that the terminology and indexing within databases make searching with both controlled vocabulary and free-text terms problematic. Furthermore, the use of controlled vocabularies and indexing is not applied as rigorously across the social science databases as in medical databases (17). Consequently, additional search techniques are essential, as well as the need to search in more databases than is required for healthcare questions.

The inclusion of social services in SBU brought new perspectives to SBU’s work on the health sector, for instance, a more diversified approach to research designs other than RCT (e.g., time series designs), introducing alternative statistical measures, based on continuous measures rather than dichotomous (e.g., Standardized Mean Difference) and greater emphasis on the importance of legal aspects (e.g., consequences of social rights legislation).

In summary, with some exceptions, the procedures and routines from systematic reviews on healthcare have been successfully adapted for reviews on social services.

## International collaboration

In 1993, SBU had a leading role in the establishment of an international organization for health technology assessment (HTA), with the formation of the International Network of Agencies for Health Technology Assessment (INAHTA) (18). SBU is still an active member of INAHTA, which is the only network for public agencies. The experiences gained from this international collaboration have been of great value to SBU’s international work in promoting a network similar to INAHTA but for organizations focusing on social services.

In 2019, the Swedish Government commissioned SBU to initiate a similar network for social services, to avoid duplication of work and to improve the quality of systematic reviews (19). There was a need for a network of publicly funded not-for-profit organizations which assess social interventions. In November 2021, the International Network for Social Intervention Assessment, INSIA, was instigated (20). Among the founding members was SBU, also honored to host their first secretariat. The other founding members are the Haute Autorité de Santé (HAS) in France, the Institut National d‘Excellence en Santé et en Services Sociaux (INESSS) in Canada, the National Institute for Health and Care Excellence (NICE) in the United Kingdom, and the Norwegian Institute of Public Health (NIPH). External interest in this initiative has been constant since the start, despite the challenges of establishing an international network during a pandemic. Among the first activities of INSIA were the formation of three working groups; one in information retrieval, one in health economics, and one in methodology.

Under the Swedish EU Presidency in 2023, SBU hosted a 1-day SIA conference in Stockholm, together with INSIA, on “How to assess social interventions with high quality, transparency and transferability? Towards a common methodology”. It was followed by the very first face-to-face membership meeting for the network and its working groups. Currently, the network has members from Canada, Denmark, England, France, India, Norway, Sweden, and Wales. The network is based on the same values and principles as the international network of agencies for HTA.

Apart from Nordic Collaboration in HTA and INAHTA, SBU has also taken an active part in the Campbell Collaboration, the Cochrane Collaboration, the European Heads of Agencies Group (HAG), the Ensuring Value in Research Network (EViR), the GRADE Working Group and Health Technology Assessment international (HTAi).

## Impact

Impact is of great importance for those who assess interventions and is central to the very purpose of HTA. The same applies to the assessment of social services. Thus, the purposes of SIA and HTA are similar: To ensure effective care by providing input to prioritization in decision-making, both in policy and in practice (21).

Although it is difficult to verify the impact of an organization like SBU which is commissioned neither to implement nor issue guidelines nor has the opportunity to administer a control condition, there are some positive indications of the impact of SBU on social services in Sweden. For example, the national target groups show increasing interest in the work of SBU. Our workshops and training courses are sought-after, and places are rapidly filled. In addition, in academia, the national social work departments are increasingly seeking collaboration with SBU. Stockholm, the capital of Sweden has decided to promote evidence-based interventions and not to support those lacking evidence (22). Examples of interventions which are promoted are parent management training (23), treatment in Foster Care in Oregon (24; 25), and individual placement and support (26). SAVRY is a risk assessment instrument for juvenile delinquents (27); Stockholm has decided to train 200 social workers in its application.

A previous report on the impact of SBU’s reports on health technologies (28) indicates that HTA reports have had a high impact on clinical guidelines, as well as moderate or high impacts on comprehensive decisions.

## Concluding remarks

One of SBU’s strengths is the transparency of SBU processes, in combination with the extensive quality assurance of the reports. Another strength is its organizational adaptability. The inclusion of the social sector is just one of several successful adaptations. Over the years new products have been added, for example, the enquiry service to support more regional needs, disclosure of evidence gaps to avoid research waste, and policy support, which is an adaptation to the strict time requirements of government commissions.

The work of SBU is intended to facilitate decision-making, thereby serving clients’ needs and protecting their rights (e.g., through the inclusion of ethical and legal issues of relevance to the assessed intervention). In this context, some characteristics of SBU are probably more important than others. We believe that a publicly funded, organized body, separate from decision-makers, but working in close collaboration with patients/clients, is essential for ensuring independence and confidence among the end-users of SBU’s products. Our main product, the SBU assessment, apart from systematic reviews on effects, also takes into account economic, organizational, societal, legal, and ethical issues. In our experience, inclusion of these important aspects gives greater breadth to the reports.

Thus, commissioning SBU to expand from health and dental care into the discipline of social services is an example of a measure adopted by the Swedish government to establish more evidence-based social services, in policy and in practice. This has been implemented by expanding the mandate of a trusted and acknowledged agency which has been actively engaged in the field of HTA since 1987 and during that time has earned its good reputation.

## References

[1] Hessle S, Vinnerljung B. Child welfare in Sweden—An overview. Stockholm studies in social work (Vol. 15). Sweden: Stockholm University, Institute of Social Work, Stockholm University, Institute of Social Work; 1999.

[2] Sundell K. Antal individer som fick insats inom socialtjänsten under 2013 [number of persons receiving support from social services during 2013] (unpublished manuscript); 2015. Available at https://www.researchgate.net/publication/372315035_Antal_individer_som_fick_insats_inom_socialtjansten_under_2013#fullTextFileContent Accessed January 29, 2024.

[3] Bergström M, Sundell KO, Leander L, Åström T. Interventions in child welfare services: A Swedish inventory. Child Fam Soc Work. 2022. First published July 12, 2022. 10.1111/cfs.12946 Accessed January 29, 2024.

[4] Sundell K, Soydan H, Tengvald K, Anttila S. Advancing social work practice from opinion-based to evidence-based: Sweden’s Institute for evidence-based social work practice. Res Soc Work Pract. 2010;20:714–722.

[5] Tengvald T, Sundell K. Mot bättre vetande. Centrum för utvärdering av socialt arbete och Institutet för utveckling av metoder i socialt arbete, 1992 till 2009 [towards better knowledge. Center for Evaluation of Social Services and the Institute for Evidence-based social work practice 1992–2009]. 2013. Stockholm: Socialstyrelsen.

[6] Ministry of Health and Social Affairs. En samlad kunskapsstyrning för hälso- och sjukvård och socialtjänst [A comprehensive knowledge management system for health care and social services] (DS 2014:9). 2014 https://www.regeringen.se/rattsliga-dokument/departementsserien-och-promemorior/2014/03/ds-20149/. Accessed January 29, 2024.

[7] Ministry of Health and Social Affairs. Förordning med instruktion för Statens beredning för medicinsk och social utvärdering [Statute 2007:1233 Directive for the Swedish Agency for Health Technology Assessment and Assessment of Social Services]. 2007. http://rkrattsbaser.gov.se/sfst?bet=2007:1233 Accessed January 29, 2024.

[8] Swedish Agency for Health Technology Assessment and Assessment of Social Services, https://www.sbu.se/sv/metod/ Accessed January 29, 2024.

[9] Chalmers I, Glasziou P. Avoidable waste in the production and reporting of research evidence. Lancet. 2009;374:86–89. 19525005 10.1016/S0140-6736(09)60329-9

[10] James Lind Alliance. http:// jla.nihr.ac.uk/ Accessed February 29, 2024.

[11] The Swedish Agency for Health Technology Assessment and Assessment of Social Services. Support for unaccompanied children and youth—Effects, experiences and perceptions. A systematic review and assessment of social and ethical aspects. SBU Policy Support no. 294, 2018, https://www.sbu.se/294e 34081426

[12] Mensah T, Hjern A, Håkanson K, Johansson P, Jonsson AK, Mattsson T, Tranæus SVB, Östlund P, Klingberg G. Organisational models of health services for children and adolescents in out-of-home care: Health technology assessment, Acta Paediatr. 2020;109:250–257. 31483896 10.1111/apa.15002 PMC7003841

[13] SBU. Methods to prevent mental ill-health in children. Stockholm: Swedish Council on Health Technology Assessment (SBU); 2010. SBU report no 202 (in Swedish). https://www.sbu.se/en/publications/sbu-assesses/methods-to-prevent-mental-ill-health-in-children/28876792

[14] Leijten P, Gardner F, Melendez-Torres GJ, van Aar J, Hutchings J, Schulz S, Knerr W, Overbeek G. Meta-analyses: Key parenting program components for disruptive child behavior. J Am Acad Child Adolesc Psychiatry 2019;58:180–190. 30738545 10.1016/j.jaac.2018.07.900

[15] Van der Stouwe T, Asscher JJ, Stams GJ, Dekovic M, van der Laan PH. The effectiveness of Multisystemic Therapy (MST): a meta-analysis. Clin Psychol Rev. 2014;34:468–481. 25047448 10.1016/j.cpr.2014.06.006

[16] Sundell K, Olsson TM. Svenska effektutvärderingar av beteendemässiga, psykologiska och sociala insatser 1990–2019. [Swedish effectiveness research on behavioral, psychological and social interventions 1990–2019]. Stockholm: Forskningsrådet för arbetsliv, hälsa och välfärd. 2021. https://forte.se/app/uploads/2021/04/svenska-effektutvarderingar-av-beteendemassiga-psykologiska-sociala-insatser-1990-2019-forte-rapport-april-2021.pdf

[17] Papaioannou D, Sutton A, Carroll C, Booth A, Wong R. Literature searching for social science systematic reviews: consideration of a range of search techniques. Health Info Libr J. 2009;27:114–122. 10.1111/j.1471-1842.2009.00863.x 20565552

[18] INAHTA (International Network of Agencies for Health Technology Assessment). www.inahta.org. Accessed January 29, 2024.

[19] Ministry of Health and Social Affairs. Uppdrag att initiera och etablera ett internationellt nätverk för samarbete inom området social utvärdering [Commission to initiate and establish an international network for collaboration in STA], S2019/03408/FST (partly); 2019. https://www.regeringen.se/regeringsuppdrag/2019/08/uppdrag-att-initiera-och-etablera-ett-internationellt-natverk-for-samarbete-inom-omradet-social-utvardering/ Accessed: January 29, 2024.

[20] INSIA (International Network for Social Intervention Assessment). https://www.insia.network/. Accessed January 29, 2024.

[21] Health Technology Assessment. Int J Technol Assess Health Care. 2009;25:10–10. doi:10.1017/S0266462309090345 19500433

[22] Stockholm Social Welfare Committee (2022). Att utvärdera effekterna av socialtjänstens insatser. Rapportering av budgetuppdrag [Evaluating the effects of the social service’s efforts. Reporting of budget assignments] Dnr 3.1.1–573/2022. https://insynsverige.se/stockholm-soc/protokoll?date=2022-11-22 Accessed January 29, 2024.

[23] SBU. Föräldrastödsprogram vid utagerande beteende hos barn: effekter och verksamma komponenter. SBU kommenterar 2019/08 (in Swedish). Available from: https://www.sbu.se/sv/publikationer/sbu-kommentar/foraldrastodsprogram-vid-utagerande-beteende-hos-barn-effekter-och-verksamma-komponenter/

[24] Åström T, Bergström M, Håkansson K, Jonsson AK, Munthe C, Wirtberg I, et al. Treatment foster care oregon for delinquent adolescents: A systematic review and meta-analysis. Res Soc Work Pract. 2019:1049731519890394.

[25] SBU. Treatment Foster Care Oregon for seriously delinquent adolescents: A systematic review and assessment including economic and ethical aspects. Stockholm: Swedish Agency for Health Technology Assessment and Assessment of Social Services (SBU); 2018. SBU report no 279 (in Swedish). Available from: https://www.sbu.se/en/publications/sbu-assesses/treatment-foster-care-oregon-for-seriously-delinquent-adolescents/34061483

[26] SBU. Independent living programmes for adolescents in institutional care. Stockholm: Statens beredning för medicinsk och social utvärdering (SBU); 2023. SBU Enquiry Service. Available from: https://www.sbu.se/en/publications/responses-from-the-sbu-enquiry-service/independent-living-programmes-for-adolescents-in-institutional-care/

[27] SBU. Risk and needs assessment regarding reoffending in adolescents: A systematic review and assessment of medical, economic, social and ethical aspects. Stockholm: Swedish Agency for Health Technology Assessment and Assessment of Social Services (SBU); 2019. SBU report no 303 (in Swedish). Available from: https://www.sbu.se/303e 33939351

[28] Rosén M, Werkö S (2014). Does health technology assessment affect policy-making and clinical practice in Sweden? Int J Technol Assess Health Care. 2014;30:265–272. doi:10.1017/S0266462314000270. 25089933

